# Drug Trafficking Routes and Hepatitis B in Injection Drug Users, Manipur, India

**DOI:** 10.3201/eid1212.060425

**Published:** 2006-12

**Authors:** Sibnarayan Datta, Arup Banerjee, Partha K. Chandra, Pradip K. Mahapatra, Shekhar Chakrabarti, Runu Chakravarty

**Affiliations:** * ICMR Virus Unit, Kolkata, India;; † Jadavpur University, Kolkata, India;; ‡ National Institute of Cholera and Enteric Diseases, Kolkata, India.

**Keywords:** Hepatitis B virus, injection drug use, occult hepatitis B infection, genotype C, Manipur, India, Dispatch

## Abstract

Prevalence of hepatitis B genotype C in injection drug users in the northeastern Indian state of Manipur, neighboring the "Golden Triangle," correlates well with overland drug-trafficking routes, the injection drug use epidemic, and the spread of HIV. Further spread to other regions of India through mobile populations is possible.

Injection drug use is common in countries neighboring the "Golden Triangle" (Myanmar, Laos, and Thailand), known for heroin export to other countries. HIV and injection drug use outbreaks in countries neighboring the Golden Triangle, including the northeastern Indian state of Manipur, have been associated with drug-trafficking routes (*1*). Manipur shares a 358-km porous border with Myanmar. According to the National AIDS Control Organization, India (http://www.nacoonline.org), HIV infection rates among injection drug users in Manipur increased from 2%–3% in 1989 to >50% in 1991 and ≈64% in 2000. In addition, exposure rates of 100% for hepatitis B virus (HBV) and 92% for hepatitis C virus (HCV) have been detected among injection drug users in Manipur, and 95% of wives of injection drug users had HBV exposure (*2*). However, among both the users and their wives, prevalence of HBV genotypes and occult HBV infection (*3*), a known risk factor for hepatocellular carcinoma (HCC) (*4*), remained unknown.

HBV is classified into 8 genotypes, HBV/A through HBV/H, and is further divided into subgenotypes (*5*) that have a distinct geographic distribution and are associated with different disease outcomes. The geographic distribution of HBV is known to correlate with the anthropologic history of migration (*5*) and to the origin and routes of spread of HBV infection. In addition, behavioral patterns are known to change HBV genotype distribution in a region (*6*).

Manipur is an important location, where mainland India (prevalent genotypes HBV/A, HBV/D) geographically meets China and Southeast Asia (prevalent genotypes HBV/B, HBV/C). Our study was designed to detect HBV DNA among injection drug users in Manipur and to analyze HBV genotypes for correlation with injection drug use and the HIV epidemic.

## The Study

We examined HIV-positive injection drug users from Manipur who had been identified as anti-HBc–positive during previous serosurveys conducted by the National Institute of Cholera and Enteric Diseases (*2*). Serum samples (stored at –80°C) taken from 63 men 18–25 years of age were available for the study. HBsAg detection was repeated with a monoclonal antibody–based Hepanostika hepatitis B surface antigen (HBsAg) kit (bioMérieux, Marcy l'Etoile, France). Anti-HCV antibody was detected using the Ortho HCV 3.0 test (Ortho-Clinical Diagnostics, Raritan, NJ, USA). We completed HBV DNA isolation, PCR amplification, genotype/subgenotype/subtype identification, recombination detection, and HBV DNA quantification with methods described earlier (*7**–**9*). We also compared nucleotide (Table A1) and deduced amino acid sequences (Table) with consensus sequence of amino acids of corresponding genotypes to detect substitutions (GenBank accession nos. DQ356432–DQ356441).

**Table:** Serum HBV DNA levels and amino acid variability in the partial s gene, including the region encoding the major hydrophillic loop, compared with consensus sequences*

Isolate	Serum HBV DNA (×10^3^ copies/mL)	110†	113†	114†	122‡	126†	131†	134†	143†	145	159†	160‡	161†	163
Cn A	–	I	S	T	K	T	N	F	T	G	A	K	Y	W
Cn D	–	–	–	S	–	–	T	Y	S	–	G	–	F	–
Cn C	–	L	T	S	–	I	T	–	S	–	–	R	F	–
IDU1	ND	–	–	–	–	–	–	–	S	–	G	–	F	–
IDU2	ND	–	–	–	–	–	–	–	–	–	–	–	F	–
IDU3	45.0	–	–	–	–	–	T	–	S	–	–	–	F	–
IDU4	14.0	L	–	S	–	I	T	–	S	–	–	R	F	–
IDU5	1.05	L	T	S	–	I	–	–	S	–	–	–	F	–
IDU6	ND	L	T	S	–	I	T	–	S	R	–	R	F	R
IDU7	ND	L	T	S	–	I	T	–	S	R	T	R	F	R
IDU8	9.0	L	T	S	–	I	T	–	S	–	–	R	F	–
IDU9	ND	L	T	S	–	I	T	–	S	–	–	R	F	–
IDU10	64.0	L	T	S	–	I	T	–	S	–	–	R	F	–

*Indicated by Cn, followed by genotype. HBV, hepatitis B virus; IDU, injection drug user; ND, not detectable by the assay. †Genotype-specific sites. ‡Subtype-specific sites.

All those tested were HBsAg negative, and only 10 (15.9%) had detectable HBV DNA. Anti-HCV was detected in all but 1 sample (no. 4). Serum HBV DNA level was detectable in 5 of 10 samples (Table); the rest were below the detection limit of our assay.

**Figure 1:**
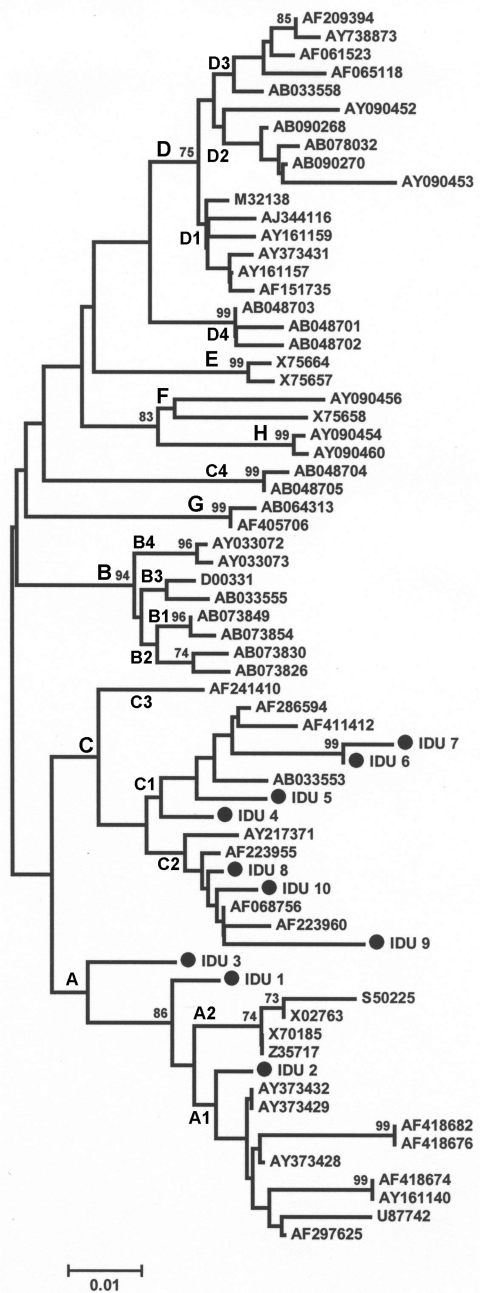
Phylogenetic relationships among the sequences of s gene from hepatitis B virus strains isolated in this study (shown with prefix "IDU") compared with reference sequences from GenBank (accession nos. are shown). Genotype and subgenotypes are indicated at each main branch and subbranch, respectively. Percentage of bootstrap replications supporting the clusters (>75%) are also shown at the nodes.

HBV/C (all subtype adr except 1 adw2) was the predominant genotype, with 4 subgenotype C1 (HBV/C1) and 3 subgenotype C2 (HBV/C2) isolates. HBV/A1 adw2 was found in 1 of the isolates. Two other isolates (nos. 1 and 3, subtype adw2) indicated a possibility of intergenotypic recombination (Figure 1), but subgenotype could not be assigned for them.

**Figure 2:**
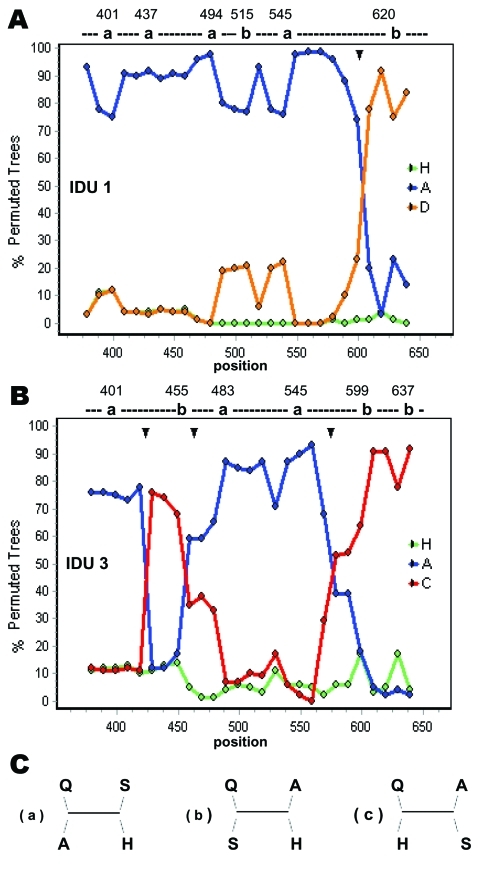
The location of recombination events in isolates IDU1 (A) and IDU3 (B), determined by using the bootscanning program of Simplot. Possible tree topologies (a, b, and c) are shown (C). The phylogenetically informative sites and the tree topologies supported at each of those sites are indicated over each plot. In the tree topologies, Q, A, and H indicate query sequences (IDU1 or IDU3), genotype A, and genotype H (outgroup) consensus sequences, respectively. S indicates consensus sequence of genotype D and genotype C for IDU1 and IDU3, respectively. Possible crossover points are indicated by solid triangles. A sliding window size of 60 bp, step size of 10 bp, Kimura-2 parameter, 1,000 bootstrap replicates, and neighbor-joining method were used for the analysis of recombination.

BLASTN search (http://www.incogen.com/public_documents/vibe/details/NcbiBlastn.html) of sequence from sample no. 1 showed similarity to HBV/D as well as to HBV/A sequences from European countries. On the other hand, sequences from sample no. 3 showed similarity to HBV/C and HBV/A sequences from Southeast Asian countries and India. Simplot analysis confirmed recombination in these 2 isolates (Figure 2). Apart from the genotype-specific substitutions, deduced amino acid sequences did not have any remarkable escape mutant other than G145R in 2 isolates (Table).

## Conclusions

The data from this study showed occult HBV infection in 15.9% of the injection drug users tested. The rate of HBV DNA detection (10%–45%) was considerably different in studies reported from different cohorts of injection drug users in different countries, a finding that has been attributed to coinfection with HCV or low HBV DNA levels (*3*). Apart from HIV, our study group had a high frequency of HCV infection and low HBV DNA levels. Undetectable HBsAg, except in 2 cases with G145R substitution, may also be a result of the above-mentioned factors.

Although the importance of occult HBV infection is not well understood, a recent study reported occult HBV to be a significant risk factor for HCC, especially among persons who were anti-HBc–positive (*4*). Another recent study among HIV-infected patients documented death due to liver disease in 22% who were HBV coinfected, 44% who were HCV/HBV coinfected, and 15% who were HBV coinfected and had HCC (*10*). Thus, the clinical relevance in our study group also needs to be followed.

Although we detected a 100% prevalence of anti-HBc in our serosurveys (*2*), only 1 was HBsAg-positive. Therefore, the distribution of genotypes among those who were HBsAg positive could not be determined.

Findings of HBV/C1 (prevalent in China) appear to support the history of human migration from China to northeastern India. However, we did not detect HBV/B, also prevalent in China. Further, we did detect HBV/C2, which has close similarity to strains from Southeast Asian countries. The presence of HBV/C correlated well with drug-trafficking routes and the injection drug use epidemic. The geographic proximity of Manipur to the Golden Triangle, needle sharing among injection drug users, and drug traders thus contributed to the spread of HBV through drug-trafficking routes, similar to HIV (1).

In Manipur, HIV subtypes C and Thai B are prevalent (*11*); these are also prevalent in the India-Myanmar and China-Myanmar border regions. The presence of similar HIV subtypes among injection drug users in Manipur supports the presence of similar HBV strains (e.g., HBV/C1, HBV/C2) and their cotransmission through drug-trafficking routes. In addition, circulation of recombinant HBV is common among injection drug users because of repeated exposure (*12*). The intergenotypic HBV recombinants found in this study are thus expected. HBV/C has been associated with advanced liver disease and poses a higher risk for HCC in Asians (*13*) than does HBV/B. However, the clinical relevance of HBV recombinants and their pathogenesis is not well understood and needs further investigation.

Poor, unemployed youths in the northeastern states of India are being recruited for drug trafficking to other regions (http://www.ipcs.org). Furthermore, national highways are associated with the prevalence of injection drug use in rural Manipur (*14*). As these highways connect Manipur with other parts of India, HBV/C may spread from Manipur to other parts of the country through persons who travel regularly, such as truck drivers and drug traffickers.

Presence of HBV genotypes A and D among patients from northern and western India is well documented. Recent research reported HBV/C with close similarity to Southeast Asian strains only from eastern India (*9**,**15*) and suggested injection drug users as a possible route of introduction (*15*). In addition, persons from northeastern India frequent Kolkata for education, employment, medical treatment, and other purposes. Studies on these mobile populations might provide further important information on the route, population at risk for infection, and changing epidemiology of these viral infections in other regions.

In conclusion, HBV/C, associated with severe liver disease in Southeastern Asia, may be emerging in the Manipur state of India through the trafficking routes of injection drugs. This genotype could spread to the general population through different modes. In light of growing information on the severity of liver disease in HBV-infected HIV/HCV patients, injection drug users should be the focus of additional education and healthcare efforts. The possibility of further spread of HIV/HBV/HCV through mobile populations to other regions of India warrants attention and further investigation.

## Appendix

**Table A1:** Nucleotide variability in the partial s gene, including the region encoding the major hydrophillic loop, compared with consensus sequences*

Isolate	367	400	414	436	451	454	457	482	484	491	493	494	499	505	511	514	519	520	529	531	544	546	555	562	574	581	586	587	592	598	616	619	629	630	633
Cn A1	T	A	T	A	T	T	A	A	T	T	C	A	A	C	A	C	A	A	G	C	C	A	T	A	A	A	T	G	T	C	A	T	G	C	A
Cn A2	–	–	–	G	–	–	–	–	–	–	A	–	–	T	–	A	–	–	–	–	–	–	–	–	–	–	–	–	–	–	G	C	–	–	–
Cn D	–	C	–	G	C	–	–	–	–	–	T	T	C	–	–	A	G	–	–	–	A	C	A	C	C	T	C	–	–	–	–	C	–	G	–
Cn C1	C	C	–	G	C	C	–	C	–	A	A	T	T	–	–	A	–	G	–	T	A	C	–	T	–	T	C	–	C	T	–	C	–	–	–
Cn C2	–	C	–	G	C	C	–	C	–	A	A	T	T	–	–	A	–	G	–	T	A	C	–	T	–	T	C	–	–	T	–	–	–	–	G
IDU1	–	–	–	–	–	–	–	–	–	–	A	–	–	T	–	A	–	–	–	–	–	–	–	–	–	T	–	–	–	–	–	C	–	G	–
IDU2	–	–	–	–	–	–	G	–	–	–	A	–	–	–	–	–	–	–	–	–	–	–	–	–	–	–	–	–	–	–	–	–	–	–	–
IDU3	–	–	–	G	C	C	–	–	–	–	A	–	–	–	–	–	–	–	–	–	–	C	–	–	–	T	C	–	–	T	–	–	–	–	–
IDU4	–	–	–	G	C	C	–	C	–	–	A	T	C	–	–	A	–	G	–	T	A	C	–	T	–	T	C	–	C	T	–	–	–	–	G
IDU5	C	–	–	G	C	C	–	C	C	A	A	T	–	–	–	A	–	G	–	T	A	–	–	C	–	T	C	–	C	T	–	C	–	–	–
IDU6	C	–	C	G	C	C	G	C	–	A	A	T	C	–	–	A	–	G	–	T	A	C	–	T	–	T	C	A	C	T	–	C	–	–	G
IDU7	C	–	C	G	C	C	G	C	–	A	A	T	C	–	G	A	–	G	–	T	A	C	–	T	–	T	C	A	C	T	–	C	A	–	G
IDU8	–	C	–	G	C	C	–	C	–	A	A	T	–	–	–	A	–	G	–	T	A	C	–	T	–	T	C	–	–	T	–	–	–	–	G
IDU9	–	C	–	G	C	C	–	C	–	A	A	T	T	–	–	A	–	G	A	T	A	C	–	T	–	T	C	–	–	T	–	–	–	–	G
IDU10	–	C	–	G	C	C	–	C	–	A	A	T	T	–	–	–	–	G	–	T	–	C	–	T	–	T	C	–	–	T	–	–	–	–	G

*Indicated by Cn, followed by genotype/subgenotype. IDU, injection drug user.
